# Influence of Anodal Transcranial Direct Current Stimulation (tDCS) over the Right Angular Gyrus on Brain Activity during Rest

**DOI:** 10.1371/journal.pone.0095984

**Published:** 2014-04-23

**Authors:** Benjamin Clemens, Stefanie Jung, Gianluca Mingoia, David Weyer, Frank Domahs, Klaus Willmes

**Affiliations:** 1 Department of Psychiatry, Psychotherapy and Psychosomatics, Medical School, RWTH Aachen University, Aachen, Germany; 2 Brain Imaging Facility, Interdisciplinary Center for Clinical Research, Medical School, RWTH Aachen University, Aachen, Germany; 3 Neurological Clinic, Section Neuropsychology, Medical School, RWTH Aachen University, Aachen, Germany; 4 Department of Psychology, Eberhard Karls University, Tübingen, Germany; 5 Knowledge Media Research Center, IWM-KMRC, Tübingen, Germany; 6 Department of Germanic Linguistics, Philipps-University Marburg, Marburg, Germany; University Medical Center Goettingen, Germany

## Abstract

Although numerous studies examined resting-state networks (RSN) in the human brain, so far little is known about how activity within RSN might be modulated by non-invasive brain stimulation applied over parietal cortex. Investigating changes in RSN in response to parietal cortex stimulation might tell us more about how non-invasive techniques such as transcranial direct current stimulation (tDCS) modulate intrinsic brain activity, and further elaborate our understanding of how the resting brain responds to external stimulation. Here we examined how activity within the canonical RSN changed in response to anodal tDCS applied over the right angular gyrus (AG). We hypothesized that changes in resting-state activity can be induced by a single tDCS session and detected with functional magnetic resonance imaging (fMRI). Significant differences between two fMRI sessions (pre-tDCS and post-tDCS) were found in several RSN, including the cerebellar, medial visual, sensorimotor, right frontoparietal, and executive control RSN as well as the default mode and the task positive network. The present results revealed decreased and increased RSN activity following tDCS. Decreased RSN activity following tDCS was found in bilateral primary and secondary visual areas, and in the right putamen. Increased RSN activity following tDCS was widely distributed across the brain, covering thalamic, frontal, parietal and occipital regions. From these exploratory results we conclude that a single session of anodal tDCS over the right AG is sufficient to induce large-scale changes in resting-state activity. These changes were localized in sensory and cognitive areas, covering regions close to and distant from the stimulation site.

## Introduction

In recent years, transcranial direct current stimulation (tDCS) became increasingly popular in the neuroscience community [1]. tDCS enables researchers to modulate brain activity in a non-invasive, painless, and stimulation polarity-dependent manner using weak, continuous electrical currents which are applied to the head via sponge electrodes [1], [2]. A crucial advantage of non-invasive brain stimulation (NiBS) techniques such as tDCS is that the specific modulation of brain activity and the concurrent measurement of psychological and/or neuroimaging outcome variables allow researchers to infer causality in brain-behaviour relationships [3]–[5]. Concerning the working mechanisms of tDCS, there is consensus that sub-threshold electrical currents modulate resting membrane potentials and spontaneous discharge rates, thereby changing cortical excitability during and up to one hour after stimulation [6]–[9]. Anodal (positively charged) tDCS increases, whereas cathodal (negatively charged) tDCS decreases excitability of the underlying cortical area, respectively depolarizing or hyperpolarizing the neural membrane [7], [8], [10], [11]. Additionally, it was shown that tDCS-induced plasticity, especially the long-term effects, depends on intracellular calcium levels, NMDA receptors, and glutamatergic synapses [12]–[15].

Previous studies revealed that tDCS enables researchers to modulate cognitive [16]–[19] and emotional processing [20]–[22]. Moreover, tDCS was successfully incorporated into the treatment of various neurological and psychiatric diseases, such as stroke [23], [24], Parkinson's disease [25], [26], depression [27]–[29], and schizophrenia [30], [31]. A rather complicated issue for studies involving tDCS is whether the applied brain stimulation actually affected and modulated brain activity, and if so how to demonstrate such an effect on a neurophysiological level. Interestingly, recent studies revealed the potential of functional magnetic resonance imaging (fMRI) to map changes of brain activity induced by tDCS [32]–[44]. Several of these fMRI studies assessed resting-state functional connectivity in search of differences between resting-state activity before and after tDCS.

Investigations of the resting brain have also become increasingly popular in recent years [45]–[47]. In a typical resting-state study, participants in the scanner are instructed to relax and not to fall asleep, either with their eyes closed or open. Several resting-state networks (RSN) have been identified, because time series of functionally related brain areas exhibit strong correlations of brain activity observed in spontaneous low-frequency fluctuations (<0.1 Hz) of blood oxygen level-dependent (BOLD) data [48]–[53]. Although the time course across the regions within a specific RSN is consistent, different RSN can be distinguished from each other because they have distinct time courses [48]. Employing either independent component analysis (ICA) or seed-based correlational techniques, different RSN have been found in the auditory [54], [55], visual [56], [57], and sensorimotor domain [58], [59]. Additionally, RSN focusing on attention systems [60], [61], executive control [60], [62], salience [60], [62], and the default mode network (DMN) [52], [63]–[65] have been identified. Moreover, recent studies began to successfully disentangle the influence of genetic variables on RSN [66]–[68].

Recently, the number of studies combining tDCS and fMRI in order to investigate resting-state activity has been increasing. Keeser and colleagues [37] found that anodal tDCS over the left prefrontal cortex and cathodal tDCS over the right supraorbital region led to changes in distinct RSN, covering areas close to and distant from the stimulating electrode. Park and colleagues [40] used seed-based correlational methods to investigate the connectivity of the left dorsolateral prefrontal cortex (DLPFC) after application of anodal tDCS to the left DLPFC and cathodal tDCS to the right supraorbital region. They found increased connectivity between the left DLPFC and several right frontal regions, whereas decreased connectivity was found close to the stimulated area [40]. However, a limitation of this and other studies stimulating frontal regions [37], [40], [41] is related to the difficulty of separating the effects of anodal and cathodal tDCS: because both electrodes were placed very close to each other, it might be difficult to disentangle the effect of the anodal and the cathodal electrode. Such a rather small distance between the two electrodes reduces functional efficacy, because the smaller the distance the more current is shunted through the scalp without affecting the brain [69].

Other studies investigated changes in resting-state functional connectivity in response to primary motor cortex (M1) or primary somatosensory cortex (SM1) tDCS. Focusing on tDCS-induced changes in the motor cortex itself, Polanía and colleagues [42] revealed that cathodal tDCS increased local connectedness of a specific region within M1 during rest, whereas anodal tDCS increased long distance functional connections in M1. In another study, Polanía and colleagues [43] demonstrated differential effects of anodal and cathodal tDCS over M1 on functional connectivity between M1 and striatal and thalamic areas. Thus, tDCS over M1 also affects resting-state functional connectivity within cortico-subcortical functional networks. For both previous studies [42], [43] the reference electrode was placed over the right supraorbital region. Amadi and colleagues [32] compared the effects of anodal, cathodal, and sham tDCS over left M1, with the reference electrode placed over the right supraorbital region, on resting-state functional connectivity. They concluded that only cathodal tDCS changed resting-state functional connectivity, modulating post-tDCS activity within the DMN and the motor RSN. Lindenberg and colleagues [38] assessed the effects of bihemispheric and unihemispheric anodal tDCS over (left) M1. Their main finding was that both bihemispheric and unihemispheric tDCS decreased resting-state functional connectivity with the right hippocampus and M1, whereas connectivity with left prefrontal cortex was increased. Sehm and colleagues [44] compared bi- and unihemispheric anodal tDCS stimulating SM1. The authors conclude that both stimulation protocols result in widespread connectivity changes: bihemispheric tDCS affected motor and prefrontal regions, whereas unihemispheric tDCS affected predominately parietal, prefrontal and cerebellar regions. For both previous studies [38], [44], the reference electrode for unihemispheric stimulation was placed over the contralateral supraorbital region. Overall, it seems that the modulatory effects of tDCS on resting-state functional connectivity strongly depend on factors such as stimulation site, stimulation duration, analysis technique, and experimental design, complicating a holistic interpretation of the results of previous studies. However, there is consensus that analysing changes in RSN in response to tDCS provides further evidence for a network-based understanding of the mechanisms underlying tDCS effects. Furthermore, it has been suggested that the combination of NiBS and resting-state measurements in particular is both reasonable and desirable, because the individual limitations of the two approaches might be avoided by combining them [70].

Therefore we suggest that it may be useful and informative to further pursue such a combined approach, in order to enhance our understanding of how the human brain at rest responds to tDCS. For the present study, we tested whether it is possible to induce changes in RSN which are detectable with fMRI, by stimulating the right angular gyrus (AG), an area known to be involved in different RSN (DMN, frontoparietal RSN). More specifically, the right AG was chosen as the stimulation site due to its involvement in RSN [52], [71], [72], semantic processing [73], orienting [74], and arithmetic fact retrieval [35], [75]. The AG is conceptualized as a higher-order brain region that integrates cross-modal information, manipulates mental information, solves familiar problems, and reorients attention to important stimuli [76]. By stimulating such a heteromodal and multifunctional region of the brain, we aim to induce large-scale changes in resting-state activity, which should affect multiple RSN. Since previous studies stimulated predominantly (sensori)-motor or prefrontal regions, an important goal of this exploratory study was to extend previous findings by examining how anodal tDCS over the parietal cortex affects functional connectivity in different RSN. We examined resting-state fMRI data acquired immediately before and after the application of 20 minutes of bipolar tDCS, with the anode over the right AG and the cathode over the contralateral supraorbital region. The experimental design used here, comprising fMRI measurements immediately before and after tDCS, was already employed successfully in previous studies [32], [40], [42]–[44], [77]. Interesting results and potentially novel insights provided by the present study can be expected because (i) we placed the stimulating electrode over the parietal (instead of the frontal or the motor) cortex, and (ii) we extended the analysis to eleven RSN. Establishing further interactions between tDCS and RSN could provide important insights into how the resting brain responds to non-invasive brain stimulation. Such results might also be important for clinical settings, considering the very convenient manner in which resting-state data can be acquired. Especially in clinical settings it is rather difficult to demonstrate that tDCS actually affects brain activity. Thus, investigating changes in RSN induced by tDCS could become an approach used to reliably corroborate brain stimulation effects in healthy participants and patients, even in the absence of a cognitively demanding task.

## Materials and Methods

### 2.1 Ethics statement

The experimental procedure was approved by the Ethics Committee of the Medical Faculty of the RWTH Aachen University Hospital (protocol number: EK 073/11).

### 2.2 Participants

Eleven healthy volunteers (all male, mean age  = 43 years; SD = 12.4 years) were recruited via public announcement. All participants had normal or corrected to normal vision, no contraindications against MR measurements, and no history of neurological or psychiatric illness. The Edinburgh Handedness Inventory [78] was used to determine handedness (mean laterality quotient (LQ) = 85.4; SD = 22.8; range = 23.7–100); applying an LQ of 80 as cut-off, 10 participants were fully right handed. All participants gave their written informed consent and received compensatory payment. All experimental procedures were performed in compliance with the latest version of the Code of Ethics of the World Medical Association (Declaration of Helsinki).

### 2.3 tDCS procedure

Stimulation was performed using a CE approved, battery-driven, constant current stimulator (NeuroConn, Ilmenau, Germany), and the current was delivered to the head via two saline-soaked, 35 cm^2^ surface sponge electrodes (5 cm×7 cm each). For stimulation of the right AG, we placed the anodal electrode over the CP4 position of the EEG 10/20 system. The rationale for the positioning of the anodal electrode was derived from the study by Herwig and colleagues [79], who determined the optimal position within the EEG 10/20 system for stimulating the AG. Accurate positioning over the CP4 point was achieved with the help of a standard 64-channel EEG cap equipped with pre-defined positions for all points of the EEG 10/20 system. As a reference electrode, the cathodal electrode was placed over the contralateral (left) supraorbital area. In comparison to other montages involving extra-cephalic reference electrodes, the proposed montage with the reference electrode placed at the contralateral supraorbital area represents an optimal solution for both the experimenter and the participant [80]. Sham tDCS was not acquired due to the following reasons: first, a recent tDCS-fMRI study clearly demonstrated that sham tDCS does not induce changes in RSN [40]. Second, including a sham condition would have resulted in a very long experimental procedure, which would have been too demanding for participants.

During tDCS, participants were engaged in a simple calculation verification task in order to provide equal cognitive input for all participants. A detailed description of this task is provided elsewhere [35]. Presenting all participants with the same task during tDCS has the advantage of reducing variability of cognitive processes during stimulation: if every participant has to solve the same task, it is unlikely that each participant exhibits diverging cognitive activity during tDCS. For 20 minutes, a constant current of 2 mA intensity was delivered. The current density never exceeded 0.0517 mA/cm^2^, which is considered safe for human brain tissue [13], [81]. Current intensity was ramped up over a period of 10 seconds at the beginning and gradually faded out for 10 seconds at the end of the stimulation period. This procedure is known to decrease adverse sensations for the participant as much as possible [13]. At the end of each tDCS session, discomfort ratings were recorded to explore potential adverse effects due to electrical stimulation using a visual analogue scale comparable to the Wong-Baker Pain Rating Scale [82].

### 2.4 Image acquisition

The fMRI measurements were performed at the RWTH Aachen University Hospital, employing a Siemens 3T Trio scanner (Siemens AG; Erlangen, Germany) equipped with a 12-channel head matrix coil. Foam pads were used for immobilization of participants' heads. Each participant was scanned twice, immediately before and after the application of tDCS, and each session contained one functional run (6 minutes and 9 seconds long). The second resting-state measurement was acquired within 30 to 40 minutes after the stimulation was started. During resting-state measurements participants saw a black screen. They were instructed to relax and keep their eyes open without falling asleep, which was confirmed immediately after the scanning session. None of the participants reported having fallen asleep during the resting-state measurement. 205 functional images were acquired during the functional run, using a spin-echo EPI sequence with the following acquisition parameters: TR = 1800 ms, TE = 28 ms, flip angle  = 72°, FOV = 192×192 mm^2^, matrix size  = 64×64, 30 transversal slices, voxel size  = 3×3×4 mm^3^, interleaved scanning acquisition, gap  = 0.48 mm. High-resolution anatomical images were obtained for each participant using an MPRAGE sequence with the following acquisition parameters: TR = 2300 ms, TE = 2.98 ms, flip angle  = 9°, FOV = 256×256 mm^2^, 176 sagittal slices, voxel size  = 1×1×1 mm^3^. Total scanning time was approximately 20 minutes per session and the anatomical scan was always performed at the end of the first scanning session.

### 2.5 Image processing

SPM8 (Institute of Neurology, London, UK; www.fil.ion.ucl.ac.uk/spm) was used for pre-processing as well as later voxel-wise statistics, whereas FSL MELODIC (FMRIB, University of Oxford, UK; www.fmrib.ox.ac.uk/fsl/melodic2/index.html) was used for ICA. The first 5 volumes of each functional time series were discarded from pre-processing to prevent artefacts from transient signal changes at the beginning of each functional run. To correct for movement artefacts, a least-squares approach and a 6-parameter rigid body spatial transformation were employed for realignment of functional images. A two-pass procedure was used to register functional images to the mean image after the first realignment. We inspected all movement parameters visually and checked that none of the eleven participants exceeded the predefined movement limits of 1.5 mm, or 1.5° in either direction. Subsequent within-subject registration between functional and anatomical images was performed with the functional images as a reference image. Applying the tissue probability maps of the ICBM (International Consortium for Brain Mapping) template, the co-registered anatomical images were segmented, aligned with an atlas space, corrected for inhomogeneities, and classified into grey matter, white matter, and cerebrospinal fluid. These data were then registered to MNI space using affine transformations. Finally, functional images were re-sampled to 2×2×2 mm^3^ resolution using sinc interpolation, and spatial smoothing was applied using an 8 mm FWHM Gaussian kernel.

### 2.6 Probabilistic ICA and automatic extraction of RSN

We applied ICA using the “single-session ICA” MELODIC algorithm implemented in FSL. The probabilistic ICA (pICA) implemented in MELODIC enables the assignment of significance p-values to spatial maps [83]. Functional data were divided into a set of spatially independent maps, each with internally consistent temporal dynamics characterized by a specific time course [84]. An advantage of pICA is that it provides z-scores (i.e., intensity values), or in other words a measure of the contribution of a time course of a specific component to the measured signal within a voxel. The resulting spatial maps can be viewed as the result of a multiple regression model, enabling the user to create voxel-wise maps (for distinct networks) of quantitative measures of resting-state functional connectivity. In the following, we will replace the term ‘resting-state functional connectivity’ with the term ‘activity’ for convenience and better legibility.

For each individual participant, all functional images of a run were concatenated across time to create a single 4D image. This 4D image was then analysed using the “single-session ICA” model implemented in MELODIC [85]. A high-pass filter (>0.009 Hz) was applied to remove low frequency drifts, and a low-pass filter (<0.18 Hz) was applied to remove cardiac and breathing-related artefacts. The software was set to output 41 components, since this number of components represents one fifth of the number of functional volumes acquired. Components were estimated using the Laplace approximation of the Bayesian model evidence.

For identifying RSN we employed a method that was originally proposed by Greicius and colleagues and has been used successfully also by other researchers [66], [86]–[90]. This method automatically determines the most consistent RSN, based on an assessment of the similarity of predefined RSN-templates and the individual components [86], [87]. The templates included the following RSN: DMN, task-positive network (TPN), executive control, two frontoparietal networks (left- and right-lateralised), sensorimotor, cerebellar, auditory, and three visual networks. The TPN was obtained from the study by Fox and colleagues [61], and all other templates were derived from the study by Smith and colleagues [53]. All templates were binarized, so that the RSN templates could be used as mask images. These binarized mask images were only used for component selection, whereas for all further (random effects) analyses the original data from the ICA was analysed. Using MATLAB, we then employed an automated algorithm to select the component best reflecting the respective RSN. In the course of this selection procedure, each component was paired with each template. Following the method developed by Greicius and colleagues [86], we always took the average z-score of all voxels within the template minus the average z-score of all voxels outside the template. The component with the largest difference (i.e., goodness of fit) was selected as reflecting the participant's respective RSN. This approach has two advantages; (i) the component that reflects a specific RSN best is selected automatically without any visual inspection being involved, and (ii) p-values are assigned to each voxel within the entire brain, allowing for the calculation of voxel-wise statistics.

### 2.7 Random effects (RFX) group analyses

Recently, it was shown that in-scanner head motion can be a confounding factor in resting-state analyses [91]–[93]. To account for this source of nuisance variability and to verify that the degree of in-scanner head motion did not differ between the two scanning sessions, we used the six motion parameters estimated during realignment to calculate the framewise displacement (FD), a standard metric for quantifying mean motion per scan. FD was calculated using the formula given in the original publication by Power and colleagues [91]. This procedure resulted in one FD value for each scan, for each participant. After calculating all FD values for each participant we averaged FD values for each scanning session and compared mean FD values for each scan (pre-tDCS vs. post-tDCS) using paired t-tests. The significance level was adjusted using Bonferroni correction, to account for the high number of multiple comparisons (200 separate tests). Additionally, we averaged the FD values across all scans for each participant and computed the means of the two scanning sessions for these average values. These average FD values were also compared between the two scanning sessions, using a paired t-test.

We used SPM8 to compare different RSN before and after the application of tDCS. In a first step of the RFX analyses, the ICA-derived components representing the respective RSN were pooled into a second level analysis for all participants at an uncorrected voxel-level threshold of *p*<0.001. This pooling process resulted in statistical maps, which were used as inclusive masks. The purpose of these masks was to limit all further comparisons to those voxels, which – based on the total cohort of all participants – are significantly involved in the respective RSN. Such an approach for masking subsequent contrasts was already employed in previous studies [66], [86], [90]. More specifically, for each RSN the specific contrast (e.g. pre-tDCS>post-tDCS) was masked inclusively with the contrast defining the respective RSN in all participants, i.e., (pre-tDCS+post-tDCS>0). Thus, for each RSN we only report group differences for areas actually falling within the respective RSN obtained here. Finally, we calculated voxel-wise comparisons of activity within each RSN before and after tDCS, applying two-sample t-tests as implemented in SPM8. To correct for multiple comparisons we set a cluster-level threshold of *p*<0.05 (FDR corrected) for all results reported here. All resulting statistical maps were visualized (i.e., superimposed) on the MNI 152 template brain provided in MRIcro GL [94] (http://www.mccauslandcenter.sc.edu/mricrogl/).

## Results

First, it should be noted that all participants tolerated 20 minutes of 2 mA tDCS well. No serious side effects were reported by any of the participants. However, at the beginning of the stimulation all participants reported a light, well-tolerable itching sensation under the area covered by the electrodes.

### 3.1 fMRI results

In order to check that the degree of head motion did not differ between the two scanning sessions, we calculated FD as outlined by Power and colleagues [91]. When comparing mean FD values for each of the 200 separate scans between the two scanning sessions, no significant differences were found using paired t-tests with Bonferroni correction (the significance level was adjusted to *p*<0.00025) for multiple testing (all individual *p*>0.005). We also compared the means for the two different scanning sessions after averaging FD values across all scans for each participant. No significant difference was found with the paired t-test (*p* = 0.504, *t*(10) = −0.693). These results indicate that in-scanner head motion was not significantly different between the two scanning sessions. Therefore, we suggest that this factor did not significantly affect the results presented here.

We detected at least one component consistent with (i.e., resembling) the RSN templates provided in the literature [53], [61] for each participant. When comparing RSN activity before and after the application of tDCS, we found significant changes in seven out of eleven RSN. A detailed overview of the results is presented in Table 1. In the following paragraphs we will describe the results more specifically.

**Table 1:** Overview of fMRI results.

RSN	pre-tDCS>post-tDCS	post-tDCS>pre-tDCS
**Cerebellar**	-	RH thalamus & amygdala, LH thalamus & caudate nucleus
**DMN**	RH superior occipital gyrus/cuneus (BA 18, 19)	LH superior medial gyrus/superior frontal gyrus/medial frontal gyrus (BA 8, 9, 32)
**TPN**	-	RH angular gyrus/superior parietal lobe (BA 7, 40)
**Executive Control**	-	RH superior frontal gyrus/superior medial gyrus (BA 8, 9)
**RH Frontoparietal**	RH putamen/lentiform nucleus, LH fusiform & lingual gyrus (BA 18, 19)	-
**Sensorimotor**	-	LH middle & inferior occipital gyrus/middle temporal gyrus (BA 19, 39), RH postcentral gyrus/superior parietal lobe (BA 5, 7), RH superior/middle frontal gyrus (BA 6)
**Visual (medial)**	-	RH lingual/calcarine/superior occipital gyrus (BA 17, 18)
**Auditory**	-	-
**LH Frontoparietal**	-	-
**Visual (occipital)**	-	-
**Visual (lateral)**	-	-

The table summarizes all results for the eleven RSN and the two contrasts of interest. For both contrasts, increased activity is displayed at a cluster-level threshold of *p*<0.05 (FDR corrected). (*BA =  Brodmann area; DMN =  default mode network; LH =  left hemisphere; RH =  right hemisphere; RSN =  resting-state networks; tDCS =  transcranial direct current stimulation; TPN =  task positive network*).

### 3.2.1 Cerebellar RSN

**Figure 1:**
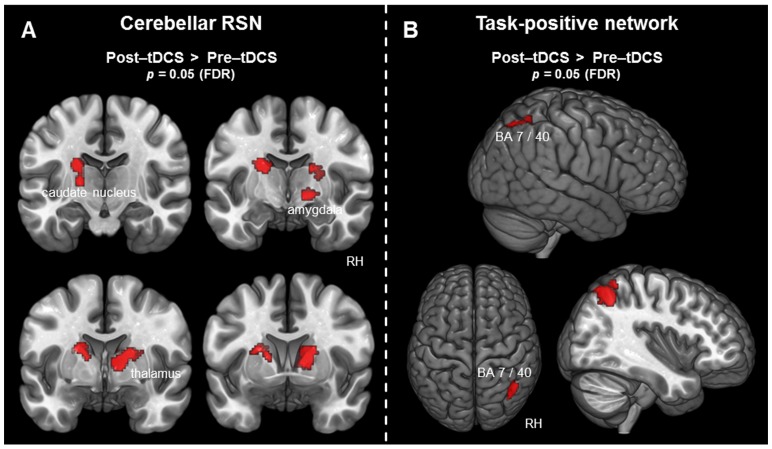
Voxel-wise difference maps for the cerebellar RSN and the TPN. Activity detected in the context of the cerebellar RSN and the TPN is displayed at a cluster-level threshold of *p*<0.05 (FDR corrected) and projected on the MNI template brain (ICBM 152). A) Areas associated with the cerebellar RSN exhibiting increased activity following tDCS. B) Areas associated with the TPN exhibiting increased activity following tDCS. (*BA =  Brodmann area; FDR =  false discovery rate; RH =  right hemisphere; RSN =  resting-state network*).

Significantly increased activity in the cerebellar RSN was found only for the contrast (post-tDCS>pre-tDCS). Increased activity after tDCS was found in the bilateral thalamus and in the right amygdala. The left caudate nucleus was also covered by the thalamic cluster of activity. The peak activation for the left cluster was located at the ventral lateral thalamic nucleus. All results for the cerebellar RSN, including MNI coordinates, t-statistics, and p-values for peak voxels of all activated clusters, are summarized in Table 2 and visualized in Figure 1A.

**Table 2:** Group differences in the cerebellar RSN, TPN, and DMN.

Anatomical Region	BA	X	Y	Z	*t* - statistic	*p* - value	No. of voxels
**Cerebellar RSN – post-tDCS>pre-tDCS**							
L thalamus/caudate nucleus		−22	−12	20	4.15	<0.001	281
R thalamus/amygdala		14	−10	−6	3.83	0.001	523
**Task positive network – post-tDCS>pre-tDCS**							
R angular gyrus/superior parietal lobe	7/40	44	−66	58	4.5	<0.001	356
**Default mode network – pre-tDCS>post-tDCS**							
R superior occipital gyrus/cuneus	18/19	14	−82	28	3.78	0.001	293
**Default mode network – post-tDCS>pre-tDCS**							
L superior medial/superior frontal/medial frontal gyrus	8/9/32	−6	26	46	5.49	<0.001	1056

All x, y, and z coordinates according to the MNI coordinate system (ICBM 152); *t*-statistics and *p*-values correspond to the peak voxels within the anatomical region(s) specified in the left column. For all contrasts, increased activity is reported at a cluster-level threshold of *p*<0.05 (FDR corrected). (*BA =  Brodmann area; L =  left hemisphere; R =  right hemisphere; RSN =  resting-state network; tDCS =  transcranial direct current stimulation*).

### 3.2.2 Task positive network (TPN)

For the TPN, significantly increased activity was found only after tDCS in the contrast (post-tDCS>pre-tDCS). The cluster of activity, as visualized in Figure 1B, was located at the right AG, covering Brodmann area (BA) 40. Additionally, the activated cluster also covered the superior parietal lobe (SPL) at BA 7. An overview of the results for the TPN, including MNI coordinates, t-statistic, and p-value for the peak voxel of the activated cluster, is given in Table 2.

### 3.2.3 Default mode network (DMN)

**Figure 2:**
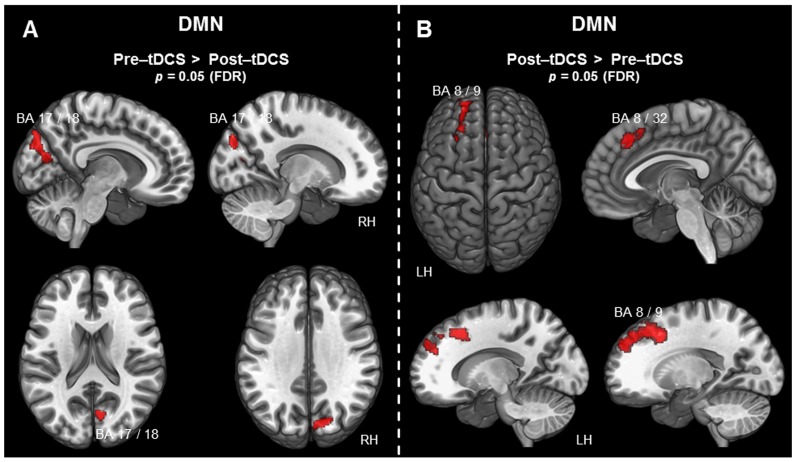
Voxel-wise difference maps for the DMN. Activity detected in the context of the DMN is displayed at a cluster-level threshold of *p*<0.05 (FDR corrected) and projected on the MNI template brain (ICBM 152). A) Areas associated with the DMN exhibiting decreased activity following tDCS. B) Areas associated with the DMN exhibiting increased activity following tDCS. (*BA =  Brodmann area; DMN =  default mode network; FDR =  false discovery rate; LH =  left hemisphere; RH =  right hemisphere*).

The contrast (pre-tDCS>post-tDCS) revealed decreased activity following tDCS in the right superior occipital gyrus (SOG). This right SOG cluster covered BA 17, BA 18, and BA 19, extending towards the cuneus (see Figure 2A). The reverse contrast (post-tDCS>pre-tDCS) revealed increased activity following tDCS in a large left frontal cluster. This cluster included the superior frontal gyrus (SFG), superior medial gyrus (SMG), and the medial frontal gyrus (meFG), as can be seen in Figure 2B. Overall, it included lateral as well as medial portions of BA 8, BA 9, and BA 32. All results for the DMN, including MNI coordinates, t-statistics, and p-values for peak voxels of all activated clusters, are summarized in Table 2 and visualized in Figure 2.

### 3.2.4 Right frontoparietal RSN

**Figure 3:**
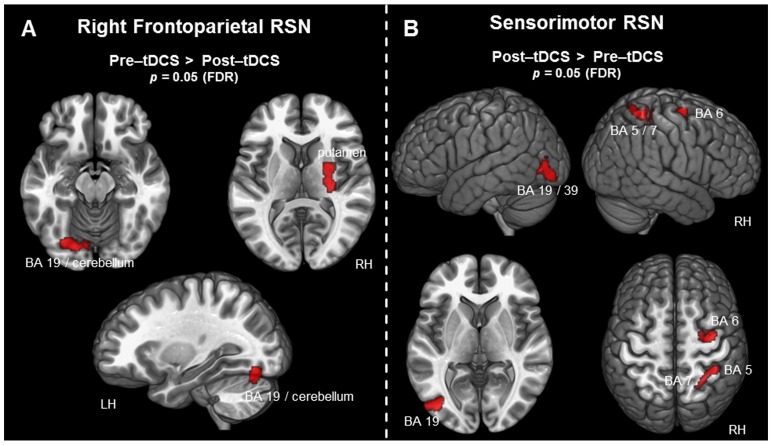
Voxel-wise difference maps for the right frontoparietal and the sensorimotor RSN. Activity detected in the context of the right frontoparietal and the sensorimotor RSN is displayed at a cluster-level threshold of *p*<0.05 (FDR corrected) and projected on the MNI template brain (ICBM 152). A) Areas associated with the right frontoparietal RSN exhibiting decreased activity following tDCS. B) All areas associated with the sensorimotor RSN exhibiting increased activity following tDCS. (*BA =  Brodmann area; FDR =  false discovery rate; LH =  left hemisphere; RH =  right hemisphere; RSN =  resting-state network*).

For the right frontoparietal RSN, the contrast (pre-tDCS>post-tDCS) revealed decreased activity after tDCS in the right putamen, also covering the lentiform nucleus. Additionally, the left fusiform gyrus showed decreased activity after tDCS. This left occipital activation spanned BA 18 and BA 19, covering also the lingual gyrus and the cerebellum (lobule VI). All results for the right frontoparietal RSN, including MNI coordinates, t-statistics, and p-values for peak voxels of all activated clusters, are summarized in Table 3 and can be seen in Figure 3A. The contrast (post-tDCS>pre-tDCS) did not reveal any significant results for the right frontoparietal RSN.

**Table 3:** Group differences in the right frontoparietal RSN, sensorimotor RSN, executive control RSN, and medial visual RSN.

Anatomical Region	BA	X	Y	Z	*t* - statistic	*p* - value	No. of voxels
**Right frontoparietal RSN – pre-tDCS>post-tDCS**							
L fusiform/lingual gyrus	18/19	−26	−76	−16	5.92	<0.001	333
R putamen/lentiform nucleus		−26	−18	12	5.48	<0.001	495
**Sensorimotor RSN – post-tDCS>pre-tDCS**							
L middle & inferior occipital gyrus/middle temporal gyrus	19/39	−38	−74	12	6.04	<0.001	679
R postcentral gyrus/superior parietal lobe	5/7	28	−52	64	3.87	<0.001	513
R superior/middle frontal gyrus	6	28	−6	66	4.32	<0.001	265
**Executive Control RSN – post-tDCS>pre-tDCS**							
R superior frontal gyrus/superior medial gyrus	8/9	20	54	42	5.55	<0.001	295
**Medial Visual RSN – post-tDCS>pre-tDCS**							
R lingual gyrus/calcarine gyrus/superior occipital gyrus	17/18	16	−84	−2	3.61	0.001	264

All x, y, and z coordinates according to the MNI coordinate system (ICBM 152); *t*-statistics and *p*-values correspond to the peak voxels within the anatomical region(s) specified in the left column. For all contrasts, increased activity is reported at a cluster-level threshold of *p*<0.05 (FDR corrected). (*BA =  Brodmann area; L =  left hemisphere; R =  right hemisphere; RSN =  resting-state network; tDCS =  transcranial direct current stimulation*).

### 3.2.5 Sensorimotor RSN

For the sensorimotor RSN, the contrast (post-tDCS>pre-tDCS) revealed increased activity following tDCS in the left middle and inferior occipital gyrus (BA 19), in the right SFG and middle frontal gyrus (miFG) (BA 6), as well as in the right postcentral gyrus (BA 5). This postcentral cluster of activity extended in the posterior direction towards the SPL at BA 7. Furthermore, the left occipital cluster extended into the middle temporal gyrus at BA 39. No significant results were found using the contrast (pre-tDCS>post-tDCS). For an overview of all results of the sensorimotor RSN, including MNI coordinates, t-statistics, and p-values for peak voxels of all activated clusters, see Table 3 and Figure 3B.

### 3.2.6 Executive control RSN

**Figure 4:**
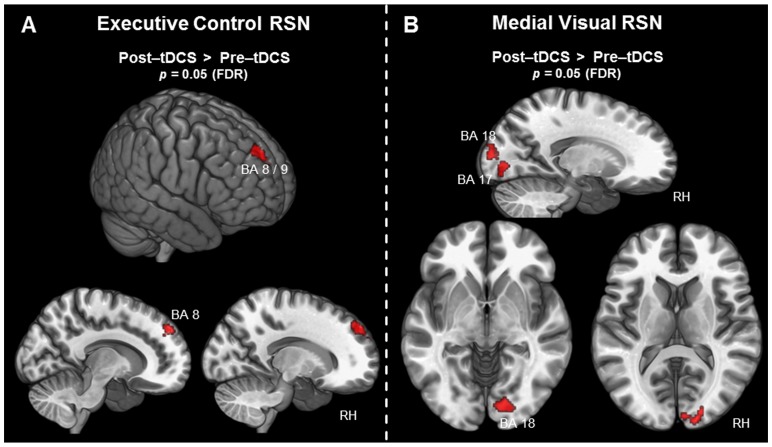
Voxel-wise difference maps for the executive control and the medial visual RSN. Activity is displayed at a cluster-level threshold of *p*<0.05 (FDR corrected) and projected on the MNI template brain (ICBM 152). A) Areas associated with the executive control RSN exhibiting increased activity following tDCS. B) All areas associated with the medial visual RSN exhibiting increased activity following tDCS. (*BA =  Brodmann area; FDR =  false discovery rate; RH =  right hemisphere; RSN =  resting-state network*).

The contrast (post-tDCS>pre-tDCS) revealed increased activity following tDCS in the right SFG for the executive control RSN. The cluster is visualized in Figure 4A. It also covers the SMG, spanning BA 8 and BA 9. While the peak activation of the cluster is clearly localized in the right hemisphere, it also covers a small portion of the SMG in the left hemisphere. For an overview of all results for the executive control RSN, including MNI coordinates, t-statistics, and p-values for peak voxels of the activated cluster, see Table 3 and Figure 4A. The contrast (pre-tDCS>post-tDCS) did not reveal any significant clusters of activity.

### 3.2.7 Visual RSN

Differences in activity before and after tDCS were found only for the first visual network, containing mostly medial visual areas [53]. The contrast (pre-tDCS>post-tDCS) did not reveal any significant results, but the contrast (post-tDCS>pre-tDCS) revealed increased activity in the right lingual gyrus. This cluster covered also the calcarine gyrus and the SOG, spanning BA 17 and BA 18. All results for the medial visual RSN, including MNI coordinates, t-statistics, and p-values for peak voxels of the activated cluster, are summarized in Table 3 and can be seen in Figure 4B.

## Discussion

The aim of the present study was to examine whether bipolar tDCS, with the anode over the right AG and the cathode over the contralateral supraorbital region, modulates the BOLD signal within any of the canonical RSN. Using methods previously validated for analysing RSN [66], [86]–[90], [95], we examined eleven RSN immediately before and after tDCS. Significant differences between the two fMRI sessions were found in seven RSN (summarized in Table 1). Following tDCS, the BOLD signal at rest was decreased in bilateral primary and secondary visual areas, and in the right putamen. Increased BOLD signal at rest following tDCS was found in several parts of the brain, including thalamic, frontal, parietal, and occipital clusters of activity.

We could induce widespread changes in several RSN following bipolar tDCS with the anode over the right AG and the cathode over the contralateral supraorbital region. In accordance with previous studies [37], [38], [40], [41], [44], [77], [96]–[98], we thus demonstrated tDCS-induced changes in distinct RSN beyond the RSN comprising the stimulation site. Concerning functional connectivity during rest, tDCS seems to induce extensive changes, not limited to the stimulation site but distributed across the whole brain. We provide both a replication and an extension of previous findings with the present study, demonstrating that also bipolar tDCS with the anode over the right AG and the cathode over the contralateral supraorbital region induces large-scale modulations of multiple RSN. Detailed, specific comparisons with previous studies are complicated by several crucial experimental parameter variations across studies concerning site, duration, intensity, and polarity of stimulation as well as analysis techniques. Nevertheless, the present results corroborate previous findings [37], [38], [40], [41], [44], [77], [96]–[98] in showing that a single session of tDCS induces complex network modulations, including but not limited to the RSN comprising the stimulation site itself, changing resting-state activity within anatomically and functionally connected brain areas. We discuss the present results more specifically in the following paragraphs.

### 4.1 Decreased resting-state activity following tDCS

We found decreased activity after tDCS for the DMN and the right frontoparietal RSN. For both networks, differences in activity were localized in the occipital cortex, covering primary and secondary visual areas. A possible explanation for the differences found in these visual areas might be that these areas are relatively close to the stimulation site, because we stimulated the right AG with a sponge electrode of 35 cm^2^. Thus, not only the AG but probably also the surrounding brain areas (e.g. secondary visual areas) were affected by tDCS, albeit to a lesser extent. Additionally, especially the right AG has been shown to exert strong functional connectivity with occipital areas [99]. Via the middle longitudinal fasciculus and the posterior part of the arcuate fasciculus, the AG is anatomically connected to several ventral temporal regions which are located in close proximity to the visual clusters of activity detected here [76], [100], [101]. Thus, changes in activity in visual areas following tDCS might be related to the fact that the AG is connected to these areas both anatomically and functionally. Regarding the fact that brain activity in occipital regions was decreased in response to anodal tDCS, it was previously shown that the application of anodal tDCS can also lead to decreased BOLD signal in areas close to the stimulation site [36], [39] during task paradigms. Future studies will have to address which stimulation parameters (localization of electrodes, intensity, duration, polarity) influence the effect of anodal tDCS on the BOLD signal, both during task paradigms and during rest.

In the context of the right frontoparietal RSN, the putamen was also less active following tDCS. It is well known that the putamen, together with other basal ganglia structures such as the caudate nucleus, is involved in motor functions, including selection, preparation, and execution of movements [102], [103]. Moreover, the putamen and other basal ganglia regions have been shown to play an important role in cognitive and emotional processing [104]–[106]. An explanation for decreased activity in the putamen might be related to connectivity between the AG and the putamen, as it was previously shown that both functional connectivity during rest and anatomical connectivity exist between the AG and the basal ganglia [107]–[109]. To some extent our findings replicate this connection between the AG and the putamen during rest and extend previous findings, by showing that stimulation of the AG also results in activity changes in the putamen during resting-state.

### 4.2 Increased resting-state activity following tDCS

Following tDCS, increased BOLD signal was found in the bilateral thalamus and the caudate nucleus. Because the AG is connected to these structures [107]–[109], increased activity might be caused by the excitability increasing stimulation induced at the AG. Although current density is highest directly underneath the stimulating electrodes, it has to be acknowledged that areas in the path of the current from the anode to the cathode will also be affected by stimulation, because the electrical field induced by tDCS is distributed across the whole brain [110]–[115]. Because electrical current will inevitably take the fastest route through the brain, possibly running along direct anatomical connections (e.g. from the AG to the thalamus), increased activity in this part of the brain may be explained by the current passing through the thalamus and the caudate nucleus on its way to the cathodal electrode. However, we can only infer the real current flow. Accurate simulations of current flow are beyond the scope of this exploratory study, but will be an interesting direction for future studies.

A similar explanation as given above may apply to the activity differences found in the left SFG and SMG, which were detected in the context of the DMN. Because the cathodal electrode was placed over the left supraorbital area, the current may have reached the left superior frontal brain regions on its way to the reference electrode. The change in activity may also be a direct effect of the cathodal electrode placed over the left supraorbital area, as it was previously shown that cathodal tDCS can lead to increased BOLD activity [34].

In the context of the TPN, we found changes of activity at the stimulation site itself. The right AG, which was directly stimulated with anodal tDCS, was found to be more active in the second fMRI session. We therefore cautiously suggest that the excitability increasing stimulation induced by anodal tDCS also increased brain activity at the stimulated area, as shown previously [35], [116]. Thus, both previous and the present results demonstrate that neuroimaging measures, such as the BOLD signal, can be increased at the stimulation site following anodal tDCS. Importantly, we also need to consider the possibility that the increased AG activity detected during resting-state might be related to a spill-over effect from the cognitive task, which was performed before the second resting-state measurement. But there was a clear difference between the pattern of activity changes detected during the task and those detected during resting-state measurements: whereas tDCS-induced changes assessed during cognitive task performance were rather focused and limited to the AG, SMA, and the retrosplenial cortex [35], we found widespread changes in many different brain areas during resting-state. Thus, we suggest that spill-over effects – if present at all – most likely affected primarily task-related activity and not the task-irrelevant resting-state activity studied here. Nevertheless, some participants may have still processed cognitive or emotional aspects related to the previously performed task during the second resting-state measurement. Participants may have for example been re-calculating or still thinking about some of the trials they could not solve, or they may have been emotionally aroused in a positive or negative manner in response to their self-perceived task performance. Such internal thought processes can distract participants away from a strict resting-state and activate brain regions related to cognitive and emotional processing. In this context, it has to be considered that such brain regions may generally be activated during a conscious resting-state, because participants might be engaged in conceptual processes involving semantic knowledge retrieval, self-awareness, and directed knowledge manipulation for organisational purposes. Thus, some of the brain regions (e.g. AG, SFG, SMG, thalamus) showing increased resting-state activity during the second measurement, which one may attribute to the modulatory effects of tDCS, might have also shown greater resting-state activity because they were involved in the aforementioned cognitive and emotional processes. Such an involvement in turn might have been influenced by the previously performed cognitive task. Thus, the arithmetic task itself might have also influenced changes in RSN. This alternative explanation for some of the present findings should be explicitly tested in future studies aimed at examining the influence of arithmetic tasks on resting-state activity, because a detailed examination of task effects on resting-state activity goes beyond the scope of the present study.

Another important aspect of the present study was that we had chosen to present participants a cognitive task during tDCS. We included this task during tDCS because we wanted to reduce the variability of cognitive processing during tDCS as much as possible. However, previous investigations have shown that on-going task activity during non-invasive brain stimulation can significantly alter stimulation-induced neuroplastic changes [117]–[120]. In 2008, Silvanto and colleagues [121] demonstrated the importance of state dependency in the context of non-invasive brain stimulation. They concluded that brain stimulation studies often do not take the current activation state of the brain into account, although the brain's response to any external stimulation (including tDCS) will be partly determined by this current activation state [121]. Thus, the brain does not react passively to external stimulation, and the effect of tDCS does not only depend on the stimulation parameters but also on the activation state of the stimulated area during the application of tDCS [122]. In accordance with these conclusions, Antal and colleagues [118] revealed that a cognitive task performed during tDCS substantially altered stimulation-induced plasticity. Interestingly, the typical pattern of increased cortical excitability following anodal tDCS and decreased cortical excitability following cathodal tDCS was reversed when tDCS over M1 was applied in combination with a cognitive task. In another study, Teo and colleagues [119] found that improvement of working memory performance was only achieved during tDCS, when stimulation and task were performed simultaneously. The authors suggested that the increased cortical excitability needed to perform the working memory task during tDCS might have resulted in a cumulative effect, leading to significant behavioural improvements only when stimulation and task were performed simultaneously. Corroborating the hypothesis of such a cumulative effect, working memory performance assessed after tDCS was not significantly improved. Furthermore, Andrews and colleagues [117] demonstrated that tDCS applied during a working memory task resulted in greater improvement of another working memory task, as compared to tDCS alone and sham tDCS applied during the working memory task. Regarding possible explanations for these findings, different authors have put forward the aforementioned hypothesis, stating that cumulative effects will be achieved if tDCS is combined with a cognitive task. Thus, task performance and simultaneous application of tDCS might lead to a greater increase in excitability than tDCS alone, which might than result in greater behavioural effects of tDCS. If task-relevant neuronal populations are already activated, they may be closer to the threshold for inducing neuroplasticity and thus are more likely to reach this threshold, if further stimulated with tDCS. Such additional task effects might induce strong synaptic activation leading to persistent strengthening of synaptic transmission, which might thus further enhance the effects of tDCS. Combining a cognitive task with tDCS could thus lead to greater neuroplastic changes, specifically in task relevant brain regions. This might also be relevant for the present study, as the AG was shown to be specifically activated by the cognitive task performed during tDCS [35]. Thus, changes in resting-state activity in the AG observed here might have been significantly enhanced by the excitability changes in this area induced by the cognitive task performed during tDCS. On the other hand, stimulation-induced neuroplastic changes might also be reversed or reduced if stimulation is combined with a cognitive task. Task-irrelevant brain regions might be deactivated, and this deactivation process might interfere with the neurophysiological processes underlying stimulation-induced neuroplastic changes [118]. Jacobson and colleagues [123] proposed that tDCS-induced neuroplastic changes can only be fully expressed if they appear in a low-competition environment, thus if participants are at rest. The authors hypothesize that if stimulated brain regions are already activated by a task, and thus in a high-competition environment, it might be more difficult to promote even further changes by introducing external stimulation. Over all, we think that the influence of cognitive task activity during tDCS represents a rather complex issue and deserves further scientific attention. For the present study, we conclude that – although we examined changes in resting-state activity only – the differences between the first and second resting-state measurement, especially in task-relevant areas such as the AG, were influenced not only by tDCS but also by the cognitive task performed during tDCS.

Because bipolar tDCS, with the anode over the right AG and the cathode over the contralateral supraorbital region, led to increased activity within several frontal and parietal areas, we suggest that the stimulation somehow increased the alert state of the brain. Consequently, this effect was best visible in areas of the brain sub-serving alertness and other attention dependent cognitive functions. For both the parietal and the aforementioned left frontal cluster, there is broad evidence that these brain areas are involved in various attention demanding tasks [74], [124]–[126]. However, the AG sub-serves other functions as well, such as verbally mediated mathematical processing [35], [75] and semantic processing [73]. Nevertheless, we cautiously conclude that the modulatory effect seen in frontal and parietal areas is due to modulation of the attentional state of the brain induced by our stimulation paradigm. This interpretation is in agreement with a previous study examining RSN in response to tDCS [37]. A similar explanation might also apply to changes found in the executive control RSN. Here, we found differences in BA 8 and BA 9 at the location of the DLPFC. The DLPFC has been linked to attentional processing numerous times and is also involved in attentional networks together with the AG [127]–[129]. Previous studies demonstrated that the DLPFC is involved in various components of attentional processing, such as reorienting, predictive coding and generation of spatially selective responses [126], [130]–[132]. We suggest that a change of the attentional state of the brain during rest, induced by bipolar tDCS, with the anode over the right AG and the cathode over the contralateral supraorbital region, led to the activity changes in this DLPFC cluster.

Overall, we found that changes in RSN were distributed across the whole brain and not limited to the stimulation sites at the right AG and the contralateral supraorbital region. This pattern of changes generally confirms previous findings [37], [38], [40], [41], [44], [77], [96]–[98], showing that connectivity between distant brain areas can be modulated using tDCS. Both the present and previous results indicate that tDCS can modulate resting-state activity in brain areas directly underneath the stimulating electrode and also in networks of functionally connected brain areas. One might thus speculate that tDCS-induced cognitive and behavioural changes reported in previous studies are not purely the result of modulated activity in a single brain region, but rather stem from a reconfiguration of different functional networks. Such a complex reconfiguration might involve changes in functional network connectivity expressed at multiple brain regions, underscoring the dynamic interplay and interactions underpinning functional networks in the human brain. The stimulation of a single brain region might thus have widely distributed effects concerning activity and connectivity of areas which are functionally and/or anatomically connected to the stimulated brain area. Such conclusions might also be important for clinical settings, in which stimulation of an appropriate cortical area might help to normalize brain activity in large-scale functional networks. However, future studies are needed to comprehensively clarify these issues and uncover the functional and behavioural relevance of such tDCS-induced changes in large-scale brain networks.

### 4.3 Limitations

We were not able to acquire brain imaging results from participants undergoing sham tDCS. Ideally, active tDCS would be compared to sham tDCS, in order to account for a potential placebo effect. Since we did not compare against sham tDCS, we cannot rule out the possibility that placebo effects (e.g. concern regarding the fact that the brain was stimulated) might in part explain the differences in brain activation observed in the present study. However, we suggest that such placebo effects most likely have the strongest impact on behavioural or peripheral physiological and not on neuro-functional measures. Providing strong support for this argument, a recent fMRI study revealed that RSN remained completely stable following sham tDCS, concluding that sham tDCS does not significantly affect RSN dynamics [41]. Furthermore, including a sham condition would have resulted in substantially longer and thus too demanding scanning times for the participants.

Another potential limitation is the relatively small sample size of the present study. However, the sample size of the present study (n = 11) is within the range of sample sizes (n = 10–25) in previous studies [32], [37], [38], [40]–[44], [77], [98] investigating this topic. In order to check whether permutation test based analyses would have changed the results obtained with the conventional parametric approach, we repeated the analyses using permutation testing, as implemented in the ‘randomise’ function of FSL. We decided to report only the results of the parametric analyses, because all findings obtained with the permutation test approach were almost identical, with none of the significant clusters of activity disappearing and no new significant clusters emerging. All of the contrasts/RSN, which did not show significant results with the parametric analyses also showed no significant results with the permutation test analyses. This indicates that our results are consistent, irrespective of the statistical methods used for analyses. Nevertheless, future studies further investigating the relationship between RSN and tDCS over the parietal cortex should aim at incorporating a sham condition and at increasing sample sizes, to allow for more powerful inferences and better generalizability.

Moreover, we would like to point out that although the experimental design with pre-and post-tDCS fMRI measurements used here might be viewed as problematic due to increased comfort with the scanner or increased fatigue towards the end of the experiment, an identical design was employed successfully several times to study tDCS-induced changes of fMRI activity [32], [40], [42]–[44], [77]. A final potential shortcoming of the present study is that there was a short time delay (10–20 minutes) between the end of the tDCS session and the start of the second fMRI measurement, because tDCS and fMRI had to be performed in different rooms with a walking distance of about 5 minutes. Additionally, we allowed participants to take a short break in order to relax and to go to the lavatory after the tDCS session. Thus, we cannot know whether tDCS effects on RSN activity would have been different, if the second fMRI measurement had been acquired immediately after tDCS. As it is known that the physiological and behavioural effects of tDCS decrease over time, one might expect that also tDCS effects on RSN activity decreased during the short time delay. Future studies might therefore reduce the time delay between brain stimulation and subsequent neuroimaging measurements, for example by applying tDCS inside the MR scanner using MR-compatible stimulators.

## Conclusions

From the results of this exploratory study we conclude that bipolar tDCS, with the anode over the right AG and the cathode over the contralateral supraorbital region, for 20 minutes results in large-scale changes of activity within several RSN. We were able to show that tDCS affected resting-state BOLD signal of the area directly underneath the stimulating electrode, namely the right AG. Several distant areas, which are connected to the AG either functionally or anatomically, also showed resting-state activity changes in response to tDCS. This might be related (i) to direct modulation in response to tDCS exerted by the AG along anatomical or functional connections or (ii) to the direct effect of the electric current, as it had to pass through these areas on its way to the cathode. Our results are corroborating previous findings [37], [38], [40], [41], [44], [77], [96]–[98], demonstrating for the first time that bipolar tDCS with the anode over the right AG and the cathode over the contralateral supraorbital region can induce both local changes underneath the stimulating electrodes as well as large-scale changes in connectivity of functional brain networks. While our exploratory results in general corroborate previous findings, a specific comparison with previous studies is complicated by considerable differences in several stimulation parameters (site, duration, intensity, and polarity) as well as data analysis techniques, which probably have an influence on tDCS-induced changes of brain activity. Overall, our exploratory study provided first evidence that a single session of bipolar tDCS with the anode over the right AG and the cathode over the contralateral supraorbital region resulted in functional changes of brain activity acquired at rest. In our previous study [35] we found task related changes only in the AG and two other regions, whereas we found widespread changes in resting-state functional connectivity distributed across the whole brain in the present study. Thus, further investigations of changes in RSN in response to non-invasive brain stimulation will be promising. However, due to the limitations outlined above, all conclusions of this exploratory study need to be interpreted cautiously and warrant further confirmation.
